# Utilisation of traditional medicine among women diagnosed with breast cancer in Ghana: a descriptive phenomenological study

**DOI:** 10.1186/s12906-024-04364-x

**Published:** 2024-01-23

**Authors:** Adwoa Bemah Boamah Mensah, Stella Baffour Asuo, Kofi Boamah Mensah, Joshua Okyere, Shalini Kulasingam, Beth Virnig, Joe-Nat Clegg-Lamptey

**Affiliations:** 1 Department of Nursing, College of Health Sciences, Kwame Nkrumah University of Science and Technology, Kumasi, Ghana; 2 Nursing and Midwifery Training College, P. O. Box F1, Fomena Adansi, Ashanti Region Ghana; 3 Department of Pharmacy Practice, Faculty of Pharmacy and Pharmaceutical Sciences, College of Health Sciences, Private Mail bag, Kwame Nkrumah University of Science and Technology, University Post Office, Kumasi, Ghana; 4 Department of Population and Health, University of Cape Coast, University Post Office, Cape Coast, Ghana; 5 Division of Epidemiology and Community Health, University of Minnesota, Minneapolis, MN USA; 6 College of Public Health and Health Professions, University of Florida, Gainesville, FL USA; 7 Department of Surgery, School of Medical Sciences, University of Ghana, Accra, Ghana

**Keywords:** Breast cancer, Utilisation, Traditional medicine, Health services, Qualitative study, Ghana

## Abstract

**Background:**

Women living with breast cancer (BC) rely on traditional medicine (TM) in addition to orthodox medicine. There is a need to understand how and why women diagnosed with BC utilise TM. This study explored and described the lived experiences of women living with BC in terms of their utilisation of traditional medicine.

**Methods:**

A descriptive phenomenology design was used to purposively conduct 20 face-to-face in-depth interviews using a semi-structured interview guide. Data were analysed using NVivo-12 based on Collaizzi’s framework for thematic data analysis.

**Results:**

Overall, five main themes emerged, namely: sources of knowledge on TM, motivations for using TM, treatment modalities, timing for the initiation of TM, the reasons for discontinuing use of TM, and the decision to seek orthodox medicine. Under the category of motivations for using TM, four themes emerged: financial difficulties and perceived cost effectiveness of TM, influence of social networks, including family and friends, assurance of non-invasive treatment, delays at the healthcare facility, and side effects of orthodox treatment. Non-invasive treatments included herbal concoctions, natural food consumption, and skin application treatments. Regarding the timing of initiation, TM was used in the initial stage of symptom recognition prior to the decision to seek orthodox medicine, and was also used complementarily or as an alternative after seeking orthodox medicine. However, patients eventually stopped using TM due to the persistence of symptoms and the progression of cancer to a more advanced stage, and disapproval by orthodox practitioners.

**Conclusion:**

Women living with BC in Ghana utilise traditional medicine (TM) for many reasons and report their family, friends and the media as a main source of information. A combination of herbal concoctions and skin application modalities is obtained from TM practitioners to treat their BC. However, they eventually discontinue TM when symptoms persist or when disapproval is expressed by their orthodox healthcare providers. We conclude that there is an opportunity to better integrate TM into the standard of oncological care for BC patients.

## Background

Globally, breast cancer (BC) remains the most common form of cancer that affects millions of women [1]. Evidence from the World Health Organisation (WHO) [2] indicates that in 2020, BC affected 2.3 million women, resulting in 685,000 fatalities worldwide; by the end of that year, the most prevalent cancer type globally was BC, with 7.8 million women diagnosed within the past five years who are still alive. A further 4.4 million cases of BC are predicted to occur worldwide by 2070 [3, 4]. Breast cancer is associated with significant psychosocial distress and trauma, which adversely affects the quality with life of those diagnosed of the disease. Consequently, there have been calls for early detection and prompt management of BC in LMIC.

Several orthodox healthcare services are employed in the management of BC among women. These include systemic therapies, endocrine therapies, targeted biologic therapy, and surgery [2]. Women diagnosed with BC have also been reported to utilise traditional, complementary and alternative medicine (TCAM), especially in LMICs [5–7]. Therefore, there is a need to understand how and why persons diagnosed of BC utilise traditional medicine (TM). However, to fully comprehend the issues associated with use of TM, it is important to first operationalise TM.

The WHO defines TM as *“the sum total of the knowledge, skill, and practices based on the theories, beliefs, and experiences indigenous to different cultures, whether explicable or not, used in the maintenance of health as well as in the prevention, diagnosis, improvement or treatment of physical and mental illness”* [8]. This study uses the definition of TM as used in the sociocultural context of Ghana, which has a more specific definition than that of the WHO. The Ghanaian understanding of TM is that it refers to *“the utilisation of plants with medicinal value as well as faith/spiritual healing for therapeutic reasons”* [9]. Available evidence has found varied levels of TM utilisation across various regions. A systematic review of the prevalence of herbal medicine use among cancer patients reported the highest use in Africa (40%) compared to other global regions [10]. In Ghana, a previous study [11] reported a high prevalence of herbal medicine use (59.2%) and faith/spiritual healing (42.9%) among cancer patients.

A plethora of studies have found factors such as age, educational status, wealth status, and marital status are significantly associated with TM utilisation among cancer patients [10, 11]. However, beyond these factors, there is a need to understand why patients diagnosed with BC choose to utilise traditional medicine. It is unclear why patients living with BC utilise TM and why they discontinue its use. Providing clarity to these issues forms the basis for this research. This study explored and described the lived experiences of women living with BC in terms of their utilisation of traditional medicine. Specifically, the study sought to explore participant’s lived experiences in respect to the:

- Sources of knowledge on the utilisation of TM in cancer treatment and management.
- Reasons for utilising TM.
- Reasons for discontinuing TM use and the factors that influence the decision to switch to orthodox medicine.

## Methods

### Study design

In this study, a descriptive phenomenological study design was employed to explore the lived experiences of patients diagnosed with breast cancer (BC) who utilise complementary therapy modalities such as Traditional Medicine (TM). Descriptive phenomenology, as outlined by Husserl [12], is a qualitative research approach that involves the systematic analysis and description of particular phenomena without imposing preconceived notions that may influence the understanding of the experiences being examined.

The philosophical underpinnings of descriptive phenomenology lie in the phenomenological reduction, a process that involves setting aside assumptions and presuppositions to explore the essence of the phenomenon as it is lived by the participants [12]. This approach aligns with the goal of our study, which is to gain an in-depth understanding of the nuanced experiences of BC patients using TM. By adopting a phenomenological stance, we aimed to capture the breadth, depth, and richness of these experiences at the time of observation, allowing for an exploration of the subjective meaning attributed to the phenomenon [12].

The decision to employ a descriptive phenomenological study design was deliberate and driven by the nature of our research objectives. Unlike other qualitative approaches, descriptive phenomenology provides a methodological framework that is particularly well-suited for exploring and unravelling the intricate details of individual experiences without imposing theoretical frameworks a priori. Moreover, descriptive phenomenological approach was chosen due to its emphasis on the first-person perspective and its ability to reveal the essence of lived experiences, aligning with the study’s focus on understanding the subjective lived experiences of BC patients utilising TM. By utilising this approach, we sought to elicit rich narratives from participants, allowing their unique perspectives to emerge organically.

### Setting

The study was conducted at the Oncological Department of Komfo Anokye Hospital (KATH), located in Kumasi, Ashanti region of Ghana.

### Population and sampling

Individuals clinically diagnosed of BC made up the target population (see Table 1). Purposive sampling was used to recruit the participants. This technique was used because we were interested in recruiting only individuals who had ever utilised TM, and could therefore contribute substantially in terms of providing rich narratives of their experiences. The eligibility criteria for prospective participants were established to maintain a balance between inclusivity and specificity. Each participant was required to be an adult, aged 18 years and above, clinically diagnosed with breast cancer, and a user of TM for their breast cancer treatment. Notably, participants were considered eligible regardless of the stage of cancer or the duration of their TM usage, thus allowing for the inclusion of individuals with varying medical histories and treatment trajectories.

### Data collection

A total of 20 face-to-face interviews were conducted using a semi-structured interview guide. The interviews were conducted by the second author (SBA) who was skilled in qualitative interviewing. The participants were given the opportunity to decide where the interviews would be held. However, all of the participants agreed to have the interviews conducted in one of the offices at the Oncology Department, KATH. Each interview was audio-recorded; additionally, field notes were taken. In each interview, probing questions (e.g., could you tell me more? Can you elaborate?) were asked to elicit detailed accounts of the experiences of the participants. The interviews lasted an average of 45 min. Interviews were conducted until the point of saturation, that is, until no new analytical information emerged. By the 17th interview, no new analytical information was emerging. However, data collection continued with three more interviews as recommended by Francis et al. [13] as a means of confirming that data saturation had been reached. The interviews were conducted in in Twi language (local dialect) and translated into English following verbatim transcription.

### Analysis

Prior to data analysis, we engaged in a reflective process to identify and acknowledge any preconceived notions or theoretical frameworks that might be introduced into the analyses. This included discussing our personal opinions about the use of TM for purposes like breast cancer treatment. Transcripts of the data collected were imported into NVivo-12 for data management and analysis. Since the study relied on a descriptive phenomenological study design, we adopted Colaizzi’s framework for thematic analysis [14]. To facilitate phenomenological reduction, the authors immersed themselves in the data through multiple readings of the transcripts. During these readings, we consciously suspended any immediate judgments or attempts to fit the data into pre-existing categories, allowing the richness of the participants’ descriptions to unfold. After this, two of the authors (ABBM and JO) conducted an independent inductive coding of the data. This approach was used because it allows the findings to emerge from the data [15]. Codes from the independent coding were compared; similar codes were identified and grouped to constitute themes and sub-themes. Patterns and interlinkages were then identified to inform the interpretation and discussion of the results. Throughout the analysis process, we engaged in regular reflexivity sessions where we openly discussed any emerging challenges. These sessions allowed us to continually reassess our approach, address potential biases, and refine our understanding of the participants’ experiences.

### Rigour

To enhance transferability and confirmability, detailed information about the study context and methodology was provided. To establish credibility, member-checking was performed with five (5) participants, one week after transcription, to verify the accuracy of the interview material. No revisions were requested by the participants, indicating that the transcripts were an accurate reflection of the interviews.

**Table 1:** Participants’ profile

Participant ID.	Age	Marital Status	Parity	Educational Background	Religion	Occupation
001	58	Married	5	No formal education	Christian	Unemployed
002	37	Married	1	JHS	Christian	Seamstress
003	48	Married	6	JHS	Christian	Hairdresser
004	47	Married	10	JHS	Muslim	Farmer
005	62	Widow	8	JHS	Christian	Unemployed
006	47	Divorced	4	JHS	Christian	Trading
007	57	Divorced	2	JHS	Christian	Farmer
008	70	Married	6	No formal education	Christian	Trading
009	74	Married	5	No formal education	Christian	Unemployed
010	54	Married	0	JHS	Christian	Trading
011	45	Married	3	No formal education	Christian	Trading
012	34	Married	3	JHS	Christian	Trading
013	49	Married	1	Form 4	Christian	Trading
014	77	Married	5	Tertiary	Christian	Pensioner
015	72	Married	9	No formal education	Christian	Unemployed
016	62	Married	4	Form 4	Christian	Trading
017	55	Married	2	Form 4	Christian	Trading
018	34	Single	2	JHS	Christian	Trading
019	36	Married	4	SHS	Christian	Trading
020	60	Married	7	Primary	Christian	Trading

## Results

**Fig. 1:**
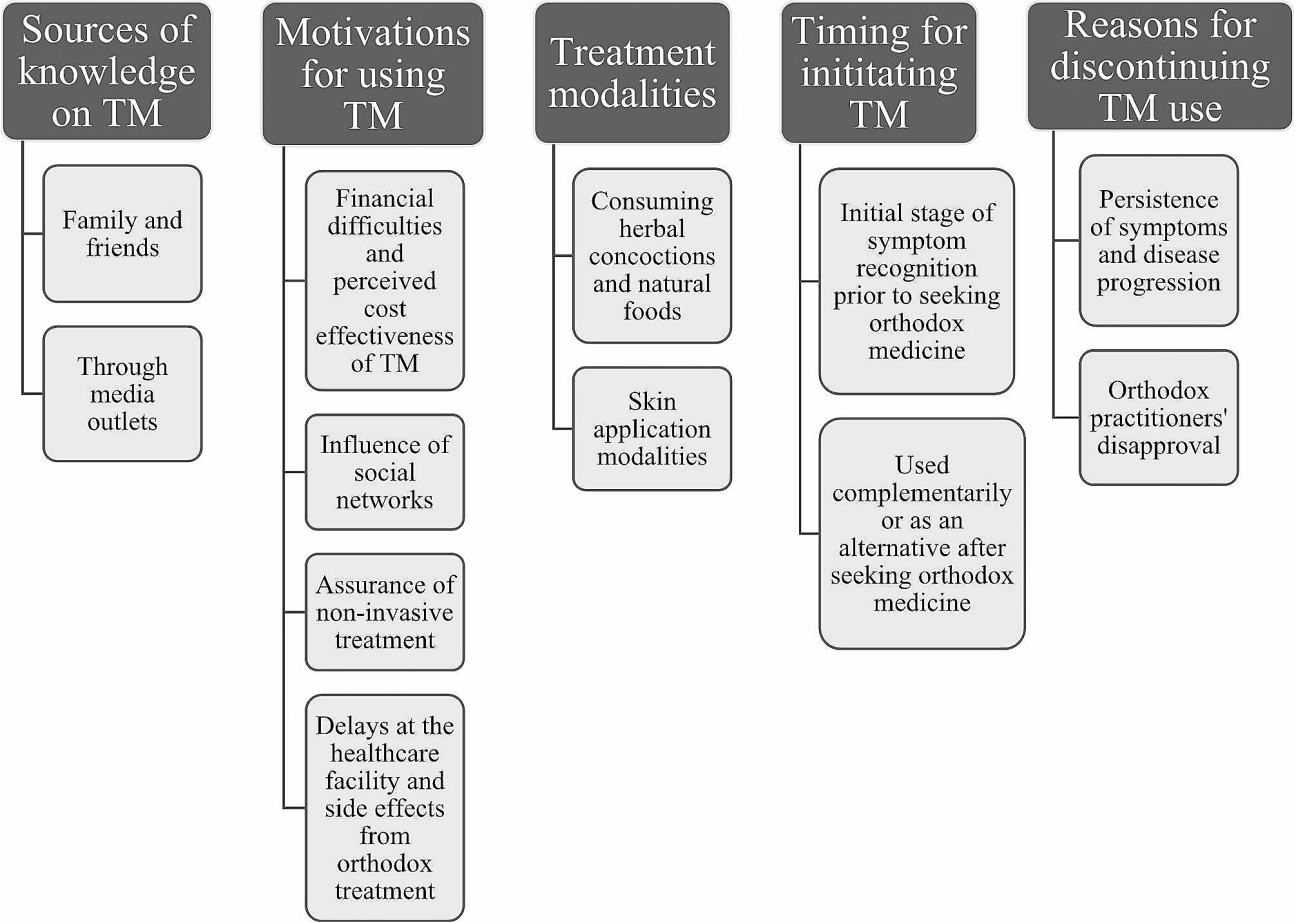
Summary of categories and themes

Overall, five main categories emerged from our thematic analysis, namely: sources of knowledge on TM, motivations for using TM, treatment modalities, timing for the initiation of TM, and the reasons for discontinuing TM use and decision to seek orthodox therapy. Under the category of motivations for using TM, four themes emerged: financial difficulties and perceived cost effectiveness of TM, influence from social networks, assurance of non-invasive treatment, delays at the orthodox healthcare facility and side effects from orthodox treatment. Herbal concoctions, natural food consumption, and skin application treatments were reported as remedies prescribed by traditional practitioners to treat BC. Regarding the timing for initiation, TM was used at the initial stage of symptom recognition prior to women deciding to seek orthodox medicine, and also used complementarily or as an alternative after seeking orthodox medicine. However, patients decided to discontinue the use of TM as a result of the persistence of symptoms and disease progression, and orthodox practitioners’ disapproval. A summary of the themes is presented in Fig. 1.

### Sources of knowledge on TM

To fully comprehend why and how persons with breast cancer utilise TM, it is imperative to gauge where they receive information from about this form of healthcare. The study revealed that the participants mainly received information through their family and friends, as well as through media outlets.

#### Through family and friends

An individual’s immediate circle of family and friends plays a critical role in their accessibility to information including health information. In this study, family and friends were one of the major sources of information and knowledge on TM. According to the participants, they trusted their family and friends. As such, any information from them was considered credible.

*“Some of my relatives have used traditional medicine for other diseases like hypertension, malaria and swelling in other parts of their bodies. And so, they were the ones who informed me about the using traditional medicine to treat my cancer”* (Participant 11).

*“I got this information from my mother and my friends who have been supporting me”* (Participant 2).

#### Through media outlets

Among the patients who participated in this study, media outlets such as the radio and community information centres were key conduits through which they received information regarding TM and its use in treating and managing breast cancer. According to the participants, media outlets often host TM and faith healer practitioners who claim to have medicines that can treat breast cancer. They discussed how TM practitioners bring people they claim to have ever treated for BC to the media outlet sponsored events to testify about the efficacy of the treatments prescribed by the TM. Given that these traditional media outlets are considered a credible source of information by Ghanaians, persons with breast cancer are convinced about the authenticity of the information received.

*“There was this herbalist on the radio who was making claims about being able to cure cancers. He even brings people to give testimonies on live radio. That was how I got to know that there was traditional medicine for my condition”* (Participant 7).

*“I got to know about the use of herbs to treat the cancer from the radio. There were some advertisements on radio and the community information centre that claimed to have herbal medications that can treat breast cancer and lumps in the breast”* (Participant 1).

### Motivations for using TM

One of the objectives of this study was to explore the reasons why women diagnosed with breast cancer utilise TM. Under this category, the following themes emerged: financial difficulties and perceived cost effectiveness of TM, influence from social networks, assurance of non-invasive treatment, as well as delays at the healthcare facility and side effects from orthodox treatment. Each of emerging themes under is discussed in detail below.

#### Financial difficulties and perceived cost effectiveness of TM

A major reason or factor that motivated participants’ use of TM was the financial difficulties that they had experienced as a result of seeking orthodox healthcare for their condition. This was mainly evident among breast cancer patients who had initiated orthodox medicine first. According to the participants, the cost of the chemotherapy and other hospital-related bills adversely affected their finances and led to an exhaustion of the individual’s savings. This made it difficult for them to continue with orthodox medicine. Therefore, opting for TM was the most cost-effective alternative for these patients:

*“That was when I was finding it difficult to get money to pay for the hospital bills and the chemotherapy. I can’t remember the exact days or months but after taking 3 chemo, I stopped because there was no money to continue. I had used up all of my savings and was no longer in employment. So, I opted for TM which was affordable”* (Participant 2).

*“I can say that one of the reasons why I initially opted for the traditional medicine was because of the cost. It didn’t cost me that much. I spent only 20ghc [$1.69] for the medicine which was way cheap when compared to what I was spending on the orthodox medicine”* (Participant 8).

Additionally, the analysis revealed that patients were concerned about other competing demands for their scarce financial resources. Hence, the high cost of seeking healthcare from the hospital or orthodox healthcare facility forced patients to make decisions that would ensure the cost-effectiveness of their treatment. This is reflected in the extract below:

*“Hmm…initially, I went for traditional medicine because of financial difficulties. Some of my kids were in boarding school so there was no money. And the traditional medicine was cheaper in terms of cost. So, I thought that would make things better”* (Participant 17).

#### Influence from social networks

Participants in this study asserted that they were encouraged by their immediate social networks (i.e., family and friends) to utilise TM. The study revealed that these social networks acted as cues that convinced the patient about the self-efficacy of TM over orthodox medicine.

*“I had faith that traditional medicine will help based on what I have heard from friends about traditional medicine. they encouraged me to use it so I did”* (Participant 19).

*“Everyone around me kept telling me that I should go for the traditional medicine because it is much effective at uprooting/curing the main cause of the disease. I was told that traditional medicine will be much effective in decreasing the size of the lump in my breast. Hence, I used it”* (Participant 6).

#### Assurance of non-invasive treatment

Another reason for the use of TM among patients diagnosed with breast cancer was the perceived assurance of non-invasive treatment modalities. The participants narrated that orthodox medicine tends to adopt invasive treatments such as the surgical removing of the breast. This sparked fear and discomfort among the participants. However, the TM practitioners and social networks that encouraged the utilisation of TM assured patients that a non-invasive approach will not lead to the loss of a body part. This assertion is embodied in the following narrations:

*“At the hospital, the doctor told me he will remove my breast so there was fear in me. However, the herbalist told me his traditional medicine will melt the lump and heal me so that my breast won’t be removed. This pushed me to rely on his herbs”* (Participant 15).

*“… he said if you have any problem with your breast, don’t go to any hospital for surgery but come to me for treatment, Because I didn’t want to undergo surgery, I decided to take up that offer from the herbalist”* (Participant 3).

Another participant shared this experience:

*“I started using traditional medicine after a doctor at Wa confirmed I have breast cancer. I was told that if I want to treat my cancer and still keep my breast, then going traditional will be best because for the hospital, they will do a surgery to remove the breast”* (Participant 8).

#### Delays at the healthcare facility and side effects from orthodox treatment

Participants of this study reported that at the healthcare facility, there were delays initiating treatment, often arising from the healthcare providers’ request for laboratory tests and scans for confirmation before initiating treatment. The perceived medical bureaucracy discouraged patients from continuing with orthodox medicine. However, TM facilities did not run such laboratory tests and scans, providing swifter healthcare services and treatment to the patient. This delay at the hospital informed the patients’ utilisation of TM:

*“There was delay…. That was when I started coming to the hospital for treatment and the doctors were not giving me any drugs. I asked them and I was told that unless I go for various scans and laboratory test to confirm the diagnosis, they cannot initiate any treatment. So, I decided to use traditional medicine for the disease….”* (Participant 13).

*“I first reported to the hospital and they started the various diagnostic processes including various scans and laboratory test. I thought the doctor will give me drugs after the scans and tests but he didn’t. He said until he confirms the diagnosis with the results, he cannot start any medications. After every scan, the doctor will tell me to go for another set of scans so I had no choice than to use traditional medicine. I wasted months waiting for lab and scan results while the disease was also progressing, the pain……, so I was pushed to use traditional medicine”* (Participant 5).

Also, the analysis revealed that participants utilised TM because of the perception and experience of side effects associated with orthodox medicine. The pain, hair loss, vomiting, loss of appetite, discomfort and other adverse health effects from chemotherapy and radiation therapy forced the patients to discontinue orthodox medicine and utilise TM instead.

*“I tried hospital treatment but the side effects from the chemotherapy and radiation therapy were too much… Pain, discomfort, vomiting, loss of appetite and even my hair going off. The suffering was so severe that I could not bear it. So, I stopped and opted for traditional medicine”* (Participant 9).

### Treatment modalities

Traditional medicine modalities vary significantly per the particular disease and TM practitioner. Therefore, the study sought to describe the nature of the treatment modalities. The analysis showed that herbal concoctions, natural foods, and skin creams and ointments constituted the treatment modalities prescribed by the TM practitioners.

#### Consuming herbal concoctions and natural foods

A recurring theme was the use of herbal concoctions and consuming healthy natural foods as treatment modalities. For participants who used herbs, they were expected to either grind it and add to their daily meals, or boil the herbs into a concoction that could be drunk for a particular period of time. The dosage and duration for taking the herbal concoction was communicated to the patient.

*“The medicine was some herb that I grinded and add to my soup or stew. So, anytime I eat, I have to add some amount of the herbs to my food”* (Participant 2).

*“It was herbal concoction. The healer told me to boil some herbs and drink it twice a day. That was what I was drinking for about three months. I drink the coke size bottle – the small (300mls) one twice a day for the three months’ period”* (Participant 3).

There were also participants who relied on naturopathic methods. That is, the reliance on healthy diet, exercise and sufficient rest as a mode of treatment for their condition.

*“Like I said, mine was like naturopathy [the use of healthy diet, exercise, and other natural methods]. So, the man gave me some natural foods like cucumber and local apple (aluguntugui) to eat so that the lump in the breast can shrink”* (Participant 11).

#### Skin application modality

The participants also reported applying herbal medications and ointments to their skin. Specifically, TM practitioners provided the patient with the medicine and/or ointments and instructed them on how to prepare and apply the medicine to the affected areas of the breast. This is reflected in the following excerpts:

*“She gave me some herbs which I had to grind, dissolve in water and then apply on my breast every morning. So, I used it as a cream. I used to rub it on the breast”* (Participant 13).

*“It was a cream that was given to me. I will grind the herb and then mix it with water until it becomes starchy like a cream. I was applying this medicine on my affected breast”* (Participant 14).

Another participant stated that:

*“The medicine I received from the herbalist was some herbs. This time around, it was not a concoction that I must drink. Rather, I was told to boil the herbs and then dip a towel or clothe into the boiled medicine, and then use it to massage the breast”* (Participant 8).

There were, however, occasions where the patient was instructed to combine both the skin application of medicines and ointments with the consumption of herbal concoctions to treat their symptoms. This is elucidated in the narrative of one of the participants: *“Yes, I used traditional medicine. For me, the herbalist gave me herbal creams to rub on my breast, and herbal concoctions to drink”* (Participant 12).

Another participant who sought care from a spiritual healer stated that she was given holy water and anointing oil. The holy water was to be drunk by the patient, while they rub the anointing oil on the affected breast: *“The pastor didn’t give me any medication apart from anointing oils and holy water. I rub the anointing oil on the breast and drunk some as well. Sometimes too I put some of the oil in water and bath with it”* (Participant 20).

### Timing of the initiation of TM use

This theme describes when the patient utilised TM. The study shows that TM was used either at the initial stage of symptom recognition prior to seeking orthodox medicine, or as complementary to or an alternative after seeking orthodox medicine.

#### Initial stage of symptom recognition prior to seeking orthodox medicine

Participants expressed that when they recognised the signs and symptoms in their breasts, they resorted to using different types of TM including herbal ointments. This was the initial response following symptom recognition. The TM was used for a period of time before resorting to orthodox medicine. The following participants shared:

*“There was a lump in the breast so I started using herbal ointment to rub the breast before going to the hospital*” (Participant 19).

*“Yes, I started taking traditional medicine from the beginning. That was in the first four months of seeing the lump in my armpit I had not started orthodox medicine yet.”* (Participant 18).

#### Used complementarily or as an alternative after seeking orthodox medicine

There were also reports of using TM as a complementary approach. That is, the patients used TM at the same time that they underwent treatment at the hospital.

*“When I came, the Gee (Komfo Anokye Teaching Hospital) doctors placed me on the cancer treatment (chemotherapy). The progress was quite slow and so my brother got some herbal medicine that I was drinking in addition to the treatment I was receiving from the hospital”.* (Participant 1).

Another participant shared that TM was used as an alternative to orthodox medicine, particularly during financial crises where the chemotherapy had exhausted the financial resources of the participant:

*“So, at the initial stage, it was strictly the hospital drugs and the chemotherapy until my money got finished before switching to the traditional medicine”* (Participant 12).

### Reasons for discontinuing TM use and decision to seek orthodox therapy

While the use of TM served the purpose of providing cost-effective, faster and non-invasive treatment and healthcare to persons with breast cancer, participants discontinued its used at some point in the disease trajectory. Primarily, the participants discontinued TM use to seek orthodox therapy due to two reasons: the persistence of symptoms and disease progression, and advice from orthodox service providers.

#### Persistence of symptoms and disease progression

A major reason for the discontinuation of TM use to seek orthodox medicine was the persistence of the recognised symptoms, and the progression of the overall condition. According to reports from the participants, they observed physical aggravation in their condition such a continuous enlargement of their breast, exacerbation of pain, and the hardening of breast lumps. The participants, therefore, decided to discontinue TM use given that their expectations were not met.

*“I stopped taking the traditional medicine because it was making the breast bigger and I believe I would have died long time if I had continued the traditional medicine looking at how bigger the breast was becoming”* (Participant 19).

Other participants stated that:

*“Oh, if the cream would help, I should have seen good results within the week, but in this case, I didn’t see any changes in my breast. I was expecting to see the lump in my breast becoming smaller or soft but nothing like that happened upon the one-week usage. Rather, the lump was becoming more painful and hardened. So, I used it for like only a week and stopped to focus on the orthodox medicine”* (Participant 4).

*“I didn’t use the medicine for long because it wasn’t helping me. The breast was becoming bigger in size so I was hoping it will reduce in size but it remained the same”* (Participant 13).

#### Orthodox practitioners’ disapproval

Participants also indicated that they received advice from their orthodox healthcare providers concerning the use of TM. Orthodox healthcare providers discouraged the participants from using TM, stating that it was going to interfere with the efficacy of the treatment being provided by the hospital.

*“My doctor discouraged me from using herbal medicine. He said that if I do that, it will interfere with my treatment and my condition will worsen. So, I listened to him and then stopped using the herbal medicine that I was taking”* (Participant 16).

*“All that he told me was that the hospital medication shouldn’t be mixed with traditional medicine. Because of that, I stopped using the traditional medicine to be able to focus the treatment from the hospital”* (Participant 18).

Another participant shared this experience: *“The doctors told me that traditional medicine can’t treat breast cancer. Besides, I didn’t see any good results at the time I was using the herbal cream.”* (Participant 4).

## Discussion

Persons living with BC relied on multiple sources for information regarding the use of traditional medicine (TM) to treat their condition. These sources included their families, friends and the media. Furthermore, our study showed that these social networks (i.e., family and friends) influenced the decision of BC patients to utilise TM. Our results are consistent with previous studies that have identified families, friends and the media as key sources of knowledge on TM use [16, 17]. A plausible explanation for this result is that BC patients are more likely to trust the information that comes from their immediate social networks rather than healthcare professionals. Moreover, these social networks provide assurance of the efficacy of non-invasive treatment that TM offers, and emphasise the cost-effectiveness of utilising TM. Also, as most healthcare professionals exhibit a negative attitude towards TM use [17], it is not surprising that sources outside of the healthcare facility were the most common sources of information on TM utilisation. This finding underscores the need for media platforms to verify the authenticity of the claims of traditional healers who advertise their TM services to BC patients through media outlets. In addition, the results regarding the sources of information on TM use emphasises the importance of family involvement in the management of BC.

One of the reasons for TM utilisation among BC patients was their current financial constraints and perceived cost-effectiveness of TM. This is corroborated by a previous qualitative study conducted in Ghana [18] that found that BC patients resort to the use of TM due to the high cost of orthodox medicine which tends to put them in a constrained financial status. Our findings align with the health belief model which states individuals are likely to use a health intervention when there is a high perceived benefit to be accrued [19]. In this case, the perceived benefit is the cost-effectiveness of TM that serves as a motivating factor for BC patients to use this form of healthcare service.

The assurance of non-invasive treatment emerged as one of the motivating factors for BC patients in Ghana to utilise TM. It is possible that BC patients prefer non-invasive treatments due to the fear of potential complications and side effects associated with invasive treatments, such as surgery (mastectomy) [20, 21]. Additionally, TM, especially herbal concoctions, are often perceived as being more natural and gentler [22], which may be a key factor that informs the healthcare seeking decisions of BC patients. This aligns with our finding of fear of the side effects of orthodox treatment as major motivating factor for TM utilisation – a result that is congruent with the work of Muhamad et al. [22]. Our findings situate within the health belief model which postulates that perceived benefits and risk are central to healthcare utilisation [19]. That is, BC patients perceive orthodox treatments as being too risky compared to TM, which offers them multiple benefits in the form of limited side effects, non-invasive alternatives at a cheaper cost than orthodoxmedicine.

Regarding the treatment modalities, the study shows that BC patients were advised to consume herbal concoctions or apply herbal medications and ointments on the affected breast. This finding is consistent with previous studies [23, 24]. The study also revealed that TM was used as the initial treatment at the time of symptom recognition, and also used in a complementary manner to orthodox medicine. The use of TM as the initial treatment at the time of symptoms recognition underscores the need to determine how best to integrate TM into the mainstream healthcare system as there will always be those who resort to the use TM first before seeking orthodox healthcare. Nevertheless, the complementary and alternative use of TM after orthodox treatment has been initiated is a signal that mainstream healthcare systems are failing BC patients. This is evident in our findings that BC patients use TM when they experience delays at orthodox healthcare facility. A similar observation has been documented in Malaysia [22] where BC patients discussed the experience of waiting for extended periods before seeing a doctor, receiving test results, or undergoing treatment. Consequently, they used this waiting time to consult traditional healers [22].

The present study also shows that BC patients discontinue TM utilisation at some point in the disease trajectory. Mainly, TM was discontinued when there was a persistence of symptoms or a progression in the overall condition. This is corroborated by other studies [25] that have found the persistence and aggravation of symptoms contributed to almost a quarter (23.9%) of cases discontinuing TM. One possible explanation for this finding is that patients may turn to TM out of desperation when orthodox medicine fails to provide the desired relief or fails to address the underlying causes of their condition [26, 27]. In such instances, TM may provide a temporary respite from symptoms or offer a sense of control over their illness. However, the persistence of the symptoms can also trigger dissatisfaction and thus, motivate BC patients to discontinue TM use.

Consistent with Kim et al.’s study [25], we found that orthodox practitioners’ disapproval of the use of TM was a factor that influenced the discontinuation of use of TM. Perhaps the disapproval from orthodox healthcare providers stems from their unfamiliarity with traditional health therapies, making it difficult for them to accept TM practices [24]. It is also possible that others may have had negative experiences with patients who used TM concurrently with orthodox therapies. Hence, explaining their disapproval of patients’ decision to use TM.

### Implications for policy and practice

This study has some implications for policy and practice. First, the study highlights the importance of family and friends as a source of information and motivating factor for TM use. Therefore, there is a need to involve the family and social networks of the patient diagnosed with BC in healthcare decision making. Also, it is imperative for media platforms to authenticate the information and claims about TM. Our findings regarding the persistence of symptoms and progression of the condition as a factor for TM discontinuation underscores the importance of integrating traditional and orthodox medicine and ensuring that patients receive appropriate information and guidance on the potential benefits and risks of both treatment options. This requires collaboration between traditional healers and orthodox practitioners to provide coordinated care and ensure that patients receive the best possible care. Overall, this study’s findings provide the evidence necessary to inform the integration of TM into the mainstream healthcare provided to persons living with BC.

### Strengths and limitations

Since the recruitment of the participants was done at the hospital rather than in the community, we were limited to only those who had ever used TM and returned for orthodox care. This means that the study does not account for the experiences of those who utilised TM without coming back to the hospital for orthodox care. The study did not include the perspectives and experiences of the traditional healers and orthodox healthcare providers. Given that the study was retrospective in nature, there is the possibility of recall bias. Notwithstanding, the reflexivity of the research team prevented us from introducing interviewer biases into the study. Also, the use of independent coding increased the validity of our study by providing a more comprehensive and nuanced understanding of the data.

## Conclusion

It is evident from our study that women living with BC in Ghana utilise TM for many reasons and have their family, friends and the media as the main sources of information. A combination of herbal concoctions and skin application modalities are commonly prescribed. Nevertheless, they discontinue TM use when symptoms persist or when disapproval is expressed by orthodox healthcare providers. We conclude that there is an opportunity to integrate TM into the mainstream oncological care for BC patients. This integration is needed because it is indicative that BC patients patronise the services of TM practitioners. Therefore, the integration could be done in a way that TM practitioners would be used a point to refer women to orthodox practitioners.

## Data Availability

The data underlying the results presented in the study are available from figshare, a public repository: 10.6084/m9.figshare.23154326.v1.

## Abbreviations

BC: Breast Cancer
LMICs: Low and middle income Countries
KATH: Komfo Anokye Teaching Hospital
TM: Traditional Medicine
